# A service evaluation of the use and outcomes of inpatient detoxification for the treatment of alcohol and opiate dependence within a community addictions service

**DOI:** 10.1192/bjo.2021.849

**Published:** 2021-06-18

**Authors:** Harry Griffin, Natasha Rishi, Mike Kelleher

**Affiliations:** Lambeth Drug and Alcohol Service

## Abstract

**Aims:**

The 2012 Health and Social Care Act transferred Addictions commissioning from the NHS to local authorities, leading to cuts of up to 30-50% of budgets and having the greatest impact on inpatient detox services. In a system with such limited capacity, effectively triaging access to detox services and optimising the efficacy of each detox has become increasingly important. NICE offers limited guidelines to assist with making these decisions, focused on assessing the severity of dependence and risk, but provides little detail on specific predictors of success. Our aim is to evaluate the nature of cases referred for inpatient alcohol or opiate detox and their treatment outcomes. This will help develop our understanding of the factors which influence achieving abstinence, and inform future decision-making regarding suitability for inpatient detox and post-detox planning. Conclusions will form part of a review of the local alcohol care pathway guidelines.

**Method:**

A retrospective case note review of all inpatient detox admissions between April 2019-March 2020 (n = 113 patients) is being undertaken. Our data collection tool extracts quantitative and qualitative data based on criteria from Alcohol use disorders (NICE, 2017), Opiate detoxification (NICE, 2019) and local pathway guidelines.

**Result:**

Preliminary analysis of data from November 2019–March 2020 (43 patients) showed that a clearly documented rationale for inpatient detox was recorded in 95% of cases. 100% of cases had a recorded AUDIT score, whilst SADQ scores were recorded in 50% of cases. 33% of cases were admitted to rehab post detox, and 19% were prescribed anti-craving medication. Abstinence at one year was confirmed in 21% of cases. 28% of clients received a second detox within one year. The rationale for inpatient detoxes in this population is to be reported.

**Conclusion:**

Preliminary data may highlight an opportunity to improve pre detox decision-making and post detox care, with confirmed abstinence in only 21% of clients at one year after detox. The low proportion of completed SADQ scores before accessing detox could offer an opportunity to improve client assessment, and the small proportion of clients prescribed anti-craving medication highlights an area of post detox care which could also be improved. The main limitation of this study is the lack of linked analysis of outcome to specific predictors, which is something that could be explored in future. It would also be valuable to gain survey data on the experience of accessing detox from a service user perspective.

